# Stability and its mechanism in Ag/CoO_x_/Ag interface-type resistive switching device

**DOI:** 10.1038/srep35630

**Published:** 2016-10-19

**Authors:** Jianbo Fu, Muxin Hua, Shilei Ding, Xuegang Chen, Rui Wu, Shunquan Liu, Jingzhi Han, Changsheng Wang, Honglin Du, Yingchang Yang, Jinbo Yang

**Affiliations:** 1 State Key Laboratory for Mesoscopic Physics, School of Physics, Peking University, Beijing 100871, P.R. China; 2 Collaborative Innovation Center of Quantum Matter, Beijing, P.R. China

## Abstract

Stability is an important issue for the application of resistive switching (RS) devices. In this work, the endurance and retention properties of Ag/CoO_x_/Ag interface-type RS device were investigated. This device exhibits rectifying *I–V* curve, multilevel storage states and retention decay behavior, which are all related to the Schottky barrier at the interface. The device can switch for thousands of cycles without endurance failure and shows narrow resistance distributions with relatively low fluctuation. However, both the high and low resistance states spontaneously decay to an intermediate resistance state during the retention test. This retention decay phenomenon is due to the short lifetime *τ (τ* = 0.5 s) of the metastable pinning effect caused by the interface states. The data analysis indicated that the pinning effect is dependent on the depth and density of the interface state energy levels, which determine the retention stability and the switching ratio, respectively. This suggests that an appropriate interface structure can improve the stability of the interface-type RS device

In recent years, due to the urgent need for high performance memories, possible new ways to fabricate nonvolatile memory have been widely explored. The resistive random access memory (RRAM), showing high storage density and high speed, can meet the requirements of the next generation memory^1^,^2^. Therefore, the fundamental physics in RRAM, namely the resistive switching (RS) effect, has been intensively studied^3^,^4^,^5^,^6^,^7^,^8^,^9^,^10^,^11^,^12^. The RS effect exists in various materials including complex oxides, such as La_0.7_Sr_0.3_MnO_3_^3^, Pr_0.7_Ca_0.3_MnO_3_^4^, and binary transition metal oxides, such as CuO_x_^5^, FeO_x_^6^, NiO_x_^7^,^8^, CoO_x_^9^,^10^, TiO_x_^11^, and HfO_x_^12^. An important issue in RS effect research is to know where the resistive switching occurs: (1) inside the functional material layers or (2) near the interface between the electrode and the functional layers. For the case (1), many studies have confirmed that the formation and rupture of localized conductive filaments in an insulating matrix should be responsible for the filament-type RS effect^13^,^14^. However, for different kinds of devices, the formation and rupture of the conductive filaments have different mechanisms, for example: electrochemical metallization mechanism (ECM), valence change mechanism (VCM), and concentration of vacancies and so on^15^. For the case (2) (the interface-type RS), several possible mechanisms have been proposed, such as electrochemical migration of oxygen vacancies^16^,^17^, trapping of the charge carriers^18^, and a Mott transition induced by the carriers doped at the interface^19^,^20^.

At present, the stability of the device has become an obstacle for the application of the RS devices. Stabilities includes endurance^21^,^22^, retention^23^,^24^, thermal stability^25^, and others. For conductive filament-type RS, many previous reports showed that the random growth of filament paths and the non-recoverable breakdown in the dielectric materials lead to a poor endurance performance. In addition, the emf voltage, chemical potential gradients, size effects and electrolyte non-stoichiometry can give rise to a chemical dissolution of the filaments, which causes a retention decay^21^,^26^. However, there are only a few papers focused on the stabilities of the interface-type RS device. The mechanism of the instability still calls for more research.

In this paper, the endurance and retention properties of the Ag/CoO_x_/Ag interface-type RS device were investigated. This device shows an excellent endurance performance, while it has a poor retention performance. It was found that the retention decay phenomenon occurs spontaneously and the reading voltage pulses can aggravate the decay process. The main features of this RS device can be well explained based on the relaxation of the metastable pinning effect in a Schottky barrier model. The possible ways to improve the stability of the interface-type RS device were proposed.

## Results

**Figure 1:**
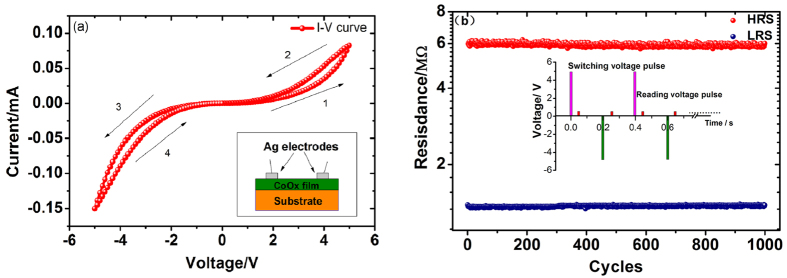
(**a**) The *I–V* curve of Ag/CoO_x_/Ag device. The inset is a schematic of the device structure. (**b**) The result of endurance test shows the HRS and LRS in a logarithmic-y-axis coordinate system. The set and reset voltages are +5 V and −5 V. The reading voltage is +0.1 V. The inset is the voltage pulse sequence applied on the device during the endurance test.

The Ag/CoO_x_/Ag RS device has been fabricated and the RS properties have been measured (see supplementary Section I for composition analysis of CoO_x_ film). A schematic of the device structure is shown in the inset of Fig. 1(a). During the *I–V* measurement, the DC voltage sweeping process can be divided into four branches: branch 1 is from 0 to +*V*_*max*_, branch 2 is from +*V*_*max*_ to 0, branch 3 is from 0 to −*V*_*max*_, and branch 4 is from −*V*_*max*_ to 0. The *I–V* curve of the device is shown in Fig. 1(a), which is a typical rectifying hysteresis *I–V* curve. This is a typical interface-type RS device base on the facts that: (1) it shows a rectifying behavior with both high and low resistance states without any abrupt switching and (2) there is an electrode area dependent resistance behavior (see supplementary Fig. S2(a)) and there are no filamentary conductive path on nanoscale (see supplementary Fig. S2(b)~(e)). Here we noticed that the *I–V* curve is non-crossing at zero voltage. This non-crossing phenomenon will be explained in the discussion section.

During the endurance test, the set and reset voltages are +5 V and −5 V, respectively, and the reading voltage is +0.1 V. The sequence of the measuring voltage pulses is shown in the inset of Fig. 1(b). The endurance with 1000 cycles is plotted in Fig. 1(b). It is obvious that there are two resistance states, namely high resistance state (HRS) and low resistance state (LRS). The switching ratio is about 500%. This indicates that a RS device based on CoO_x_ has been obtained.

**Figure 2:**
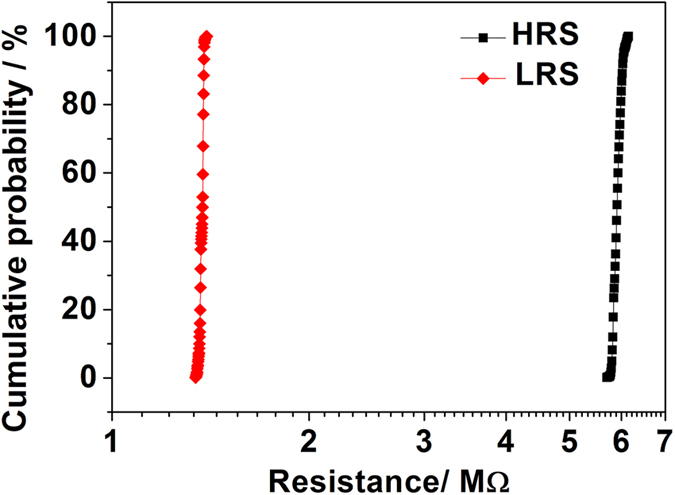
The cumulative probability of HRS and LRS distributions in the endurance test.

In order to provide a further understanding of the properties of this device, the resistance distributions of the HRS and LRS in the endurance test were investigated. It can be seen from Fig. 2 that both the resistances of HRS and LRS distribute over a narrow range. It can be seen that there are two sparse distribution regions in the curve of LRS in Fig. 2. It means that the accumulative probability increased intensively in these two regions. In other words, the distribution of LRS concentrated in these two regions. This analysis is also suitable for the distribution of HRS. That is to say, the density of data point in Fig. 2 reflects the concentration of resistance distribution. To evaluate the distribution fluctuation quantitatively, a parameter was defined as 
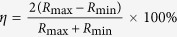
. The relative small fluctuation ratios of *η*_H_ ≈ 6.4% and *η*_*L*_ ≈ 3.0% were obtained for the HRS and LRS during the endurance test, respectively. These values indicate that the device has a good endurance performance, which is an advantage over the filament-type RS device for the data storage.

**Figure 3:**
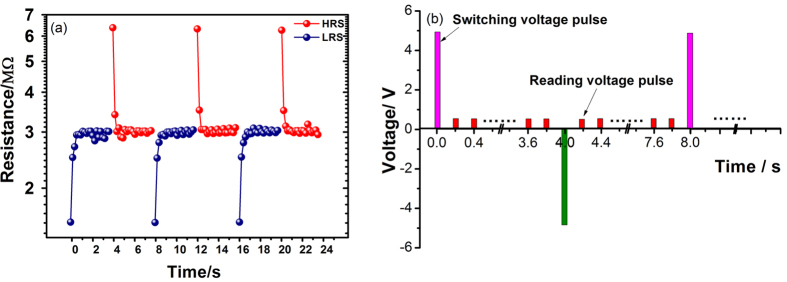
(**a**) The HRS and LRS decay quickly in the repeated reading procedure. The switching voltages are ±5 V, and the reading voltage is +0.1 V. The y-axis is in logarithmic. (**b**) The voltage pulse sequence of the repeated reading procedure.

**Figure 4:**
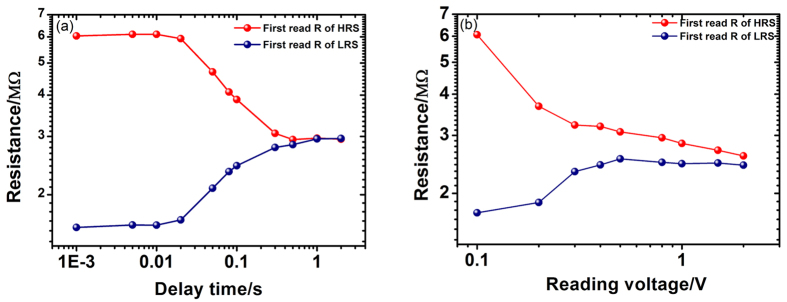
(**a**) The first read out resistances of HRS and LRS versus the delay time between the switching voltage and the first reading voltage. The switching voltages are +5 V, reading voltage is +0.1 V. (**b**) The first read out resistances of HRS and LRS versus the reading voltage. The switching voltages are ±5 V. Both (**a,b**) are in logarithmic-axis coordinate system, and they show that the resistance states decay spontaneously or under the disturbance of reading voltage pulses.

In order to study the retention property of this device, a repeated reading procedure was applied. Figure 3 plots the evolution of the resistance with the reading sequence. It was found that both the HRS and LRS decay to the same intermediate resistance state with the increase of reading times. We define the resistance difference between HRS and LRS as Δ*R* to evaluate the retention property. During the repeated reading process (see Fig. 3(a)), both the time and reading voltage pulses have strong influence on the results. In order to exclude the effect of the reading voltage, different delay times were inserted between the switching voltage pulse and the first reading voltage pulse. The result in Fig. 4(a) shows that the Δ*R* decreases with the increase of the delay time. This suggests that both the HRS and LRS decay spontaneously. The effect of reading voltage pulses was investigated by varying the reading voltages from 0.1 to 2.0 V. It is obvious that the Δ*R* decreases with the increase of reading voltage (see Fig. 4(b)). This indicates that the reading voltage pulses have a destructive effect on the HRS and LRS.

## Discussion

As it can be seen from the experiment results, this is a RS device with poor retention performance, which makes it far away from the requirements of high performance nonvolatile memories. At present, the poor stability is one of the common problems of the RS devices. For this interface-type RS, the main factors related to the stability are still not clear. In order to understand this, a phenomenological model was established in this work by taking pinning effect of interface states in consideration. This model can explain the behaviors of this interface-type RS device and was primarily verified by fitting the experiment data. During the theoretical derivation, we only consider the main process in this device to let the model be more describable than precise. Because the electric transport process in a real device is complex, and meanwhile, the most meaningful work is to provide a primary guiding on the improvement of the stability.

**Figure 5:**
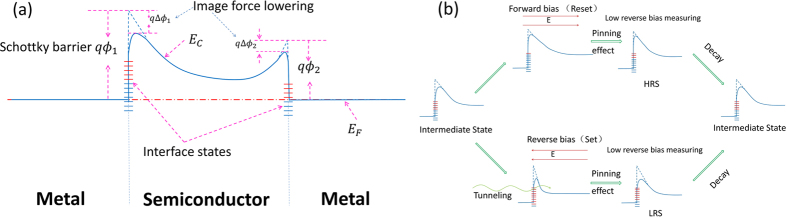
(**a**) A schematic of energy band diagram for incomplete symmetrical MSM structure, where E_c_ is the bottom of conduction band for the semiconductor, E_F_ is the Fermi energy, *qϕ*_1_ and *qϕ*_2_ are the height of Schottky barriers without image force lowering effect. *q*Δ*ϕ*_1_ and *q*Δϕ_2_ are the modifications for the barrier caused by image force lowering effect. (**b**) An illustration for the evolution process of reverse biased Schottky barrier (relative to the reading voltage) under the set and reset voltages. The different colors of the interface states represent the different filling status.

The prepared CoO_x_ film is an n-type semiconductor due to the vacancies^27^. A Schottky barrier will form at the interface between Ag metal electrode and the CoO_x_ film. This kind of metal-semiconductor junction is called Schottky barrier diode (SBD), which is also known as hot carrier diode. The structure of this device can be viewed as two SBDs connected back-to-back. With either a positive or a negative voltage applied on the device, it always has a forward biased and a reverse biased SBD in the circuit. A schematic of the initial barrier configuration in this circuit is shown in Fig. 5(a). The interface states are partially filled at the beginning. With the set or reset of external voltages, the interface states can be filled or exhausted like schematized in Fig. 5(b). After the switching process, the filling status of the interface states partially relaxed, and the unrelaxed part exhibits a pinning effect on the barrier height. However, the unrelaxed part will relax in finite time either, which causes the decay of resistance states. It means that an HRS or LRS can be obtained before the interface states relax completely.

For a theoretical model, we consider a single SBD firstly (see section III of supplementary information for the results of single SBD), the current voltage relationship should follow the equation based on the thermionic emission model^28^:


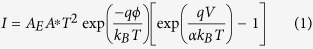


where *I* is the current, *A*_*E*_ is the area of electrode, A^*^ is the Richardson constant, *qϕ* is the Schottky barrier height at zero bias, α is the ideality factor of the Schottky barrier, *q, k*_*B*_, *T* and *V* are the charge, Boltzmann constant, temperature and applied voltage, respectively. The Schottky barrier height *qϕ* and the ideality factor α determine the basic shape of the *I–V* curve.

For a reverse biased SBD, the *V* in Eq. (1) is negative. When |*qV*| ≫ *k*_*B*_*T*, the Eq. (1) can be simplified as:


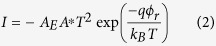


Here, we consider the image force lowering effect, which arises from the Coulomb attractive force because of the positive image charges induced inside the metal by the electrons in the conduction band of the semiconductor. The decrement of Schottky barrier caused by the image force lowering effect is sketched in Fig. 5(a). This image force lowering effect can be enhanced by a reverse bias voltage and weakened by a forward bias voltage. The decrement of Schottky barrier *q*Δ*ϕ*_*i*_ can be approximately expressed as^29^:


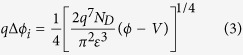


where the *N*_*D*_ and *ε* are the electrons density near the Fermi level and dielectric constant of the semiconductor, respectively. So that the Eq. (2) should be changed to:


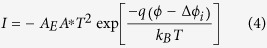


In addition, with the increase of reverse bias voltage, the thickness of the Schottky barrier will decrease. When the thickness of Schottky barrier is thinner than a threshold value *d*_*th*_, the electrons have a probability to tunnel through the barrier. This is equivalent to another lowering effect for the Schottky barrier, which can be approximately expressed as^30^,^31^:


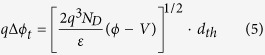


This means that the tunneling current will contribute to the current flow when the voltage drop on the reverse biased SBD exceeds a threshold value. On the other hand, the forward bias voltage will weaken the image force lowering effect. Therefore, the image force lowering effect is negligible for the forward biased SBD.

When an external voltage is applied on the device, the voltage drops on the reverse biased SBD and forward biased SBD can be denoted as −*V*_*r*_ and *V*_*f*_, respectively. The current flows *I*_*f*_ and *I*_*f*_ for the forward and reverse biased SBDs in the circuit can be expressed as:


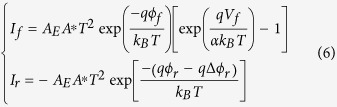


where:


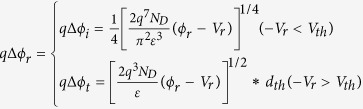


where *V*_*th*_ is the threshold voltage for the electrons to tunnel through the reverse biased Schottky barrier. As the two SBDs serially connect in the circuit, it has *I* = *I*_*f*_ = −*I*_*r*_ and *V* = *V*_*f*_ −*V*_*r*_. Here *V* is the voltage applied on the device and *I* is the current.

The Eq. (6) can be simplified with following specific situations:

(1) In the low voltage range, the voltage drop is mostly on the reverse biased SBD, *V*_*f*_ ≪*V* ≈ −*V*_*r*_<*V*_*th*_, the current can be approximated to:


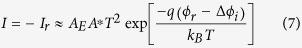


By taking logarithm, Eq. (7) can be transformed to:


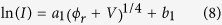


here 
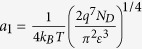
 and 
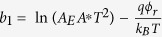
 are the transform coefficients.

(2) In the middle voltage range, *V*_*f*_ ≪ *V* ≈ −*V*_*r*_ > *V*_*th*_, the current flow is still governed by the reverse biased SBD, but the tunneling current contributes to the most of it:


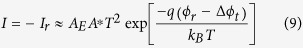


By taking logarithm, Eq. (9) can be transformed to:


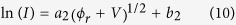


here 
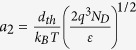
 and 
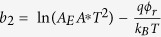
 are the transform coefficients.

In the high voltage range, *V*_*f*_ ≈ −*V*_*r*_ > *V*_*th*_, the increase of current flow increases the voltage drop on forward biased SBD. At a certain point, the forward biased SBD will dominate the current flow:


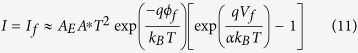


By taking logarithm and neglecting the constant 1, Eq. (11) can be transformed to:


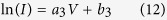


here 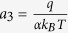 and 
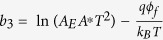
 are the transform coefficients.

As we know, there are a large number of interface states at the interface of MS junction due to an imperfect contact^32^. These interface states have a pinning effect on the Schottky barrier height since they can trap or detrap carriers^33^. The Schottky barrier can be modified by the experienced voltage. For a single SBD, the forward bias voltage decreases the *q*Δ*ϕ*_*i*_ while the reverse bias voltage increases it. The interface states can pin these modifications by trapping or detrapping the carriers. The carriers may escape from the trapped or detrapped states in a finite time, which leads to the relaxation of the pinning effect. This will result in a retention failure. The decay process should follow the decay function *e*^−λt^. Then the modification to *q*Δ*ϕ*_*i*_(*V*), caused by experienced voltage, can be expressed as a function of time t:


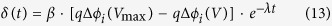


where *q*Δ*ϕ*_*i*_(*V*_max_) is the decrement of Schottky barrier at the maximum experienced voltage. Here the maximum voltage was considered to simplify the mathematic. *q*Δ*ϕ*_*i*_(*V*) is the barrier decrement at applied voltage *V* without modification. *t* is the time interval between *V*_*max*_ and *V*. λ is the decay constant, which is a parameter related to the average lifetime *τ* of the metastable states with 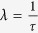. *β* is the correction coefficient, 0 ≤ *β* ≤ 1, which reflects the strength of pinning effect. Here *τ* and *β* are two constants for a device under certain environment condition.

**Figure 6:**
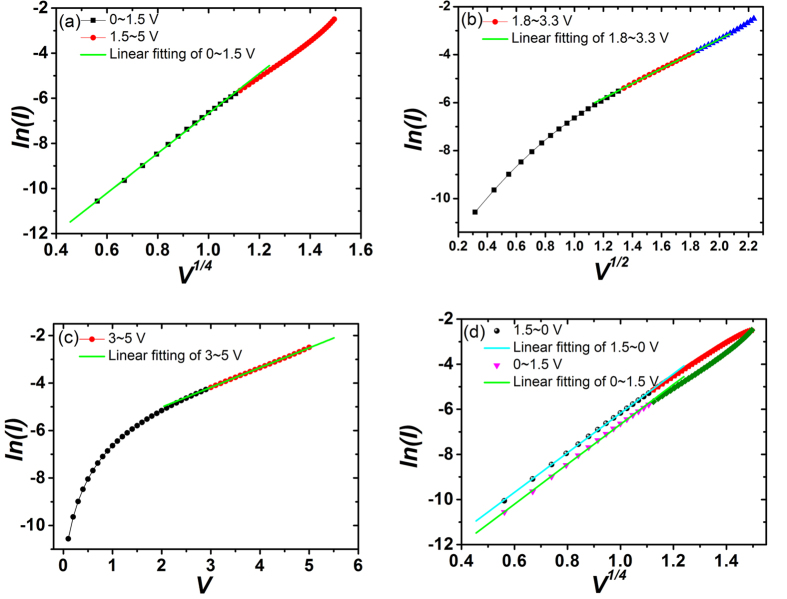
(**a–c**) are the plots of branch 1 in Fig. 1(a) with *ln*(*I*) versus *V*^*1/4*^, *V*^*1/2*^ and *V*, respectively. (**d**) The plots of branch 1 and 2 with *ln*(*I*) versus *V*^*1/4*^.

Now we can analyze the main features in our device with the theory discussed above. For the branch 1 of the *I–V* curve in Fig. 1(a) (the applied voltage increases from 0 to +5 V), the dependences of *ln*(*I*) on *V*^*1/4*^, *V*^*1/2*^ and *V* are plotted in Fig. 6(a–c), respectively. A linear dependence of *ln*(*I*) on *V*^*1/4*^, *V*^*1/2*^and *V* are observed for the ranges of 0 ≤ 1.5, 1.8 ≤ *V* ≤ 3.3, 3 ≤ *V* ≤ 5V, respectively (Two processes coexistence during the interval range and the overlap range). These relationships demonstrate that there are different origins for the current flow in different voltage ranges. In the low voltage range of 0~1.5 V, the current flow is mainly governed by the reverse biased SBD which is controlled by image force lowering effect. In the middle voltage range of 1.8~3.3 V, the tunneling current of reverse biased SBD makes the major contribution to the current flow. In the high voltage range of 3~5 V, the current flow is controlled by forward biased SBD. With the changing of voltage polarization at zero voltage, the dominant Schottky barrier is changed accordingly because the voltage drop is always concentrate on the reverse biased barrier in the low voltage range. This is the reason that the *I–V* curve in Fig. 1(a) is non-crossing at zero voltage. For the case that there is only one Schottky barrier in the circuit, the *I–V* curve will be crossing at zero voltage. This has been proved in supplementary Section III by fabricate an asymmetric-electrode device. The *I–V* curve of single SBD in Fig. S3(b) is in agreement with the *I–V* curves of asymmetric-electrode interface-type RS devices in Ref. 1and Ref. 24. This indicates that they are the same type RS devices. If the device structure is completely symmetrical, the *I–V* curve should be inversion center symmetry. However, it is difficult to prepare a completely symmetric structure, which results in an incomplete symmetric behavior in the *I–V* curve of Fig. 1(a). The schematic of incomplete symmetric band diagram is shown in Fig. 5(a). The similar analysis can be applied to the curves of branch 2. We plot the branch 1 and 2 of Fig. 1(a) with *ln*(*I*) versus *V*^*1/4*^together in Fig. 6(d). The two linear fitted lines in low voltage region are parallel, which indicates these two processes are governed by the same current mechanism. The shift of the two fitted lines is an evidence for the modification of the barrier height caused by the pinning effect from the interface states. By fitting the data in Fig. 4(a) with Eq. (7) and Eq. (13), an average lifetime *τ* ≈ 0.5 second and *β* ≈ 0.017 were obtained for Ag/CoO_x_/Ag device. Using Eq. (7) here is because that the reading voltage is in the low voltage range discussed above. The fitting curves are shown in supplementary Fig. S7(a). This is a short lifetime far away from the requirement of nonvolatile data storage but an appropriate time span to investigate the decay phenomenon.

In Fig. 1(b), the device shows different resistance values after the set and reset voltages under the same reading voltage. The evolution of the Schottky barrier in the set and reset processes is shown in Fig. 5(b). As discussed before, only the reverse biased Schottky barrier should be considered under the reading voltage of 0.1 V because it is in the low voltage range. According to Eq. (13), the +*V*_*max*_ and −*V*_*max*_ will lead to δ(0) > 0 and δ(0) < 0, respectively. These modifications can be pinned by interface states for a finite time. If we read the resistance with a low voltage in a short time after the set or reset, it will show a LRS or a HRS. However, with a longer interval time *t* to read the resistance value, the Δ*R* will decrease due to the relaxation of metastable pinning effect. The data in Fig. 4(a) can be well explained using this model. According to Eq. (13), the different *V*_*max*_ will lead to different δ(0)s. By controlling the set and reset voltages, the device will exhibit multilevel resistance states. This can be used for multibit storage, which can improve the storage density. Additionally, a lager *β* can lead to a lager |δ(0)| with the same *V*_*max*_, which gives rise to a higher switching ratio for the device.

From the above discussion, it suggests that there are two important parameters to determine the performance of the interface-type RS device, namely the lifetime *τ* and the correction coefficient *β*. Essentially, the spontaneous decay is due to the thermal fluctuation, which releases the carriers from the trapping center. In order to stabilize the RS device, one can increase the energy barrier for the trapped carriers by strain engineering or deep energy level doping. On the other hand, a larger correction coefficient *β*, corresponding to a higher switching ratio, can be achieved by increasing the density of interface states.

In conclusion, we have fabricated Ag/CoO_x_/Ag interface-type RS device and investigated its endurance and retention properties. Based on the hypothesis that the trapping and detrapping carriers of interface states are metastable, we explained the main experimental phenomena, such as rectifying hysteresis *I–V* curve, multilevel storage behavior, and retention decay phenomenon. The results indicated that the finite lifetime *τ* and the correct coefficient *β* play important roles in this kind of RS device. This may shed light on the mechanism of interface-type resistive switching.

## Methods

20 nm of Co film was deposited on SiO_2_/Si substrates by using pulsed laser deposition (PLD) employing a KrF excimer laser with a wavelength of 248 nm. The distance between target and the substrate was fixed to be 6 cm. The cobalt film was deposited at room temperature under a pressure of about 5 × 10^−8^ Torr with a laser energy density of 2 J/cm^2^ and a laser repetition rate of 5 Hz. Then the cobalt film was annealed at 550 °C for 5 minutes under 100 Torr of high purity oxygen. The chemical composition of oxygen-annealed CoO_x_ thin film was analyzed by X-ray photoelectron spectroscopy (XPS). The crystal structure of the CoO_x_ thin film was investigated using X-ray diffraction (XRD). The results of composition analysis are shown in supplementary Section I. The diameter of Ag electrodes was about ~500 *μm*. The separation distance between two electrodes was about 1 mm. All the electric measurements were done with the Keithley 2612 Source Measure Units (SMUs) at room temperature.

## Additional Information

**How to cite this article**: Fu, J. *et al*. Stability and its mechanism in Ag/CoO_x_/Ag interface-type resistive switching device. *Sci. Rep.* **6**, 35630; doi: 10.1038/srep35630 (2016).

## Supplementary Material

Supplementary Information
