# Supplementary material for: Stability and its mechanism in Ag/CoOx/Ag interface-type resistive switching device
Source: Sci Rep. 2016 Oct 19;6:35630. doi: 10.1038/srep35630 (PMC5069483; doi:10.1038/srep35630)
Supplement: Supplementary Information [file srep35630-s1.pdf]

Supplementary information

Stability and its mechanism in Ag/CoO_x/Ag interface-type resistive switching device

Jianbo Fu,[1] Muxin Hua,[1] Shilei Ding,[1] Xuegang Chen,[1] Rui Wu,[1] Shunquan Liu,[1] Jingzhi Han,[1] Changsheng Wang,[1] Honglin Du,[1] Yingchang Yang,[1] and Jinbo Yang[1, 2]

[1]State Key Laboratory for Mesoscopic Physics, School of Physics, Peking University, Beijing 100871, P.R. China

[2]Collaborative Innovation Center of Quantum Matter, Beijing, P.R. China

I. Composition analysis

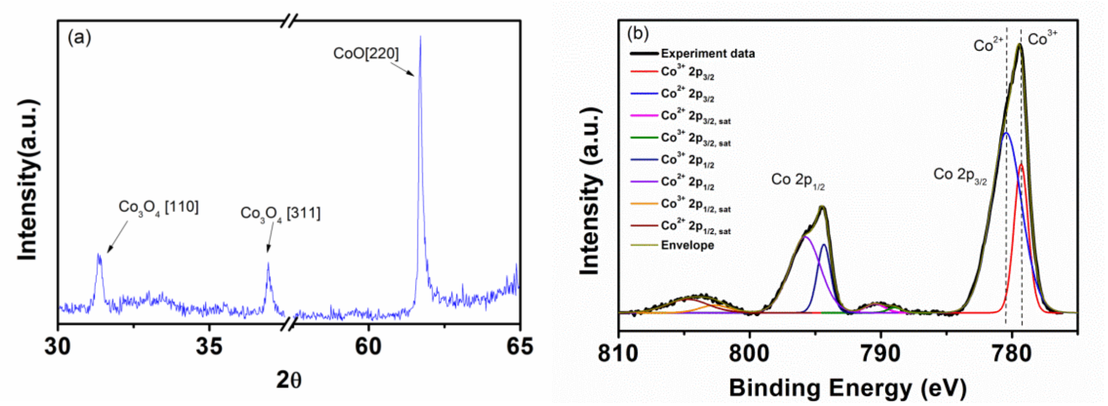

Figure S1│ (a) XRD patterns of the CoO_x thin film. (b) Co 2p electron region of XPS profile of the CoO_x thin film.

Figures S1(a) and (b) show the X-ray diffraction (XRD) patterns and X-ray photoelectron spectra (XPS) of the CoO_x film, respectively. The deconvolution of Co 2p electron region XPS profile is done with CasaXPS software. The background has been subtracted by the software automatically. As shown in Figure S1(b), Co in the surface of our sample is composed of Co^{2+} and Co^{3+} with a roughly ratio of 4:1 (mole

ratio).[1] By considering the contribution of Co_3O_4 for the Co^{2+}, a proportion (mole ratio) about 3:1 for the CoO and Co_3O_4 can be obtained from the XPS-peak-resolving analysis. We have analyzed the XPS data from three different points on the CoO_x surface and found that the proportion (mole ratio) of CoO and Co_3O_4 is almost a constant of 3:1 which shows the composition uniformity in some degree.

II. Electrode area dependent resistance and conductivity maps on nanoscale

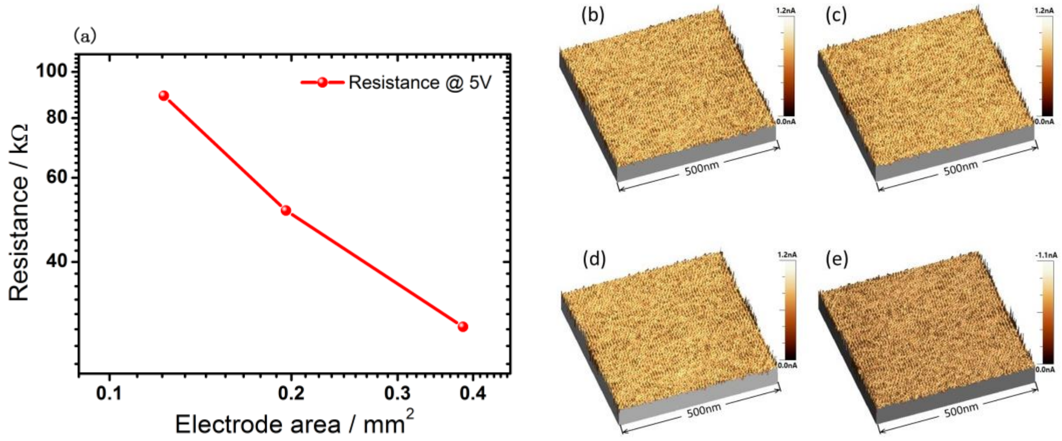

Figure S2 │ (a) The electrode area dependent resistance behavior under 5V measurement voltage. (b)~(e) The conductivity maps of areas underneath the electrodes after (b) +5V and (c) -5V switching with a probe tip bias of +5V. The conductivity maps of pristine areas without switching process with probe tip bias of (d) +5V and (e) -5V.

As demonstrated in Fig. S2(a), various diameters of 400μm, 500μm and 700μm of Ag electrodes have been fabricated by using masks. The resistances show a dependence on electrode areas. This indicates that the $Ag/CoO_x/Ag$ RS device is interface-type.[2]

In order to provide the evidence of electrical homogeneity on nanoscale, we collected the conductivity maps of the device by conductive atomic force microscope (c-AFM). The results are shown in Fig. S2(b)~(e). Generally, the conductive filaments

appear after voltage switching, namely forming process. Before scanning the conductivity maps, a forming process was realized by applying +5V or -5V voltage pulses, and then the silver paint electrodes were removed by acetone. The area covered by the electrodes (have been removed) was scanned by a +5V biased probe tip to search conductive filaments. The results are shown in Fig. S2(b) and (c). As a comparison, a pristine area without switching process was scanned with the probe tip biases of +5V and -5V, respectively, which have been shown in Fig. S2(d) and (e). As it can be seen, there are no obvious bright peaks in these four conductivity maps, which indicate there are no filamentary conductive paths on nanoscale. In addition, there is almost no difference among figures (b), (c) and (d). This is due to that the figures (b) and (c) were scanned in a long time after the voltage switching, which gives HRS and LRS enough time to decay to their intermediate states. Compared figure (e) to (d), a difference in average current intensity can be found, which is caused by the asymmetric conductivity of interface barriers. Since that the c-AFM maps in Fig. S2 have small areas relative to electrode area, we randomly scanned dozens of images on different areas to search conductive filaments (The distances among different areas are longer than at least 1 μm). The results are almost the same with the c-AFM images in Fig. S2. This can rule out the possibility of large amount filaments present in the device from nanoscale. Considering both the electrode area dependent measurements and the conductivity maps, we believed that the interface-type resistive switching governed our experiment results.

III. Asymmetric interfaces behaviors

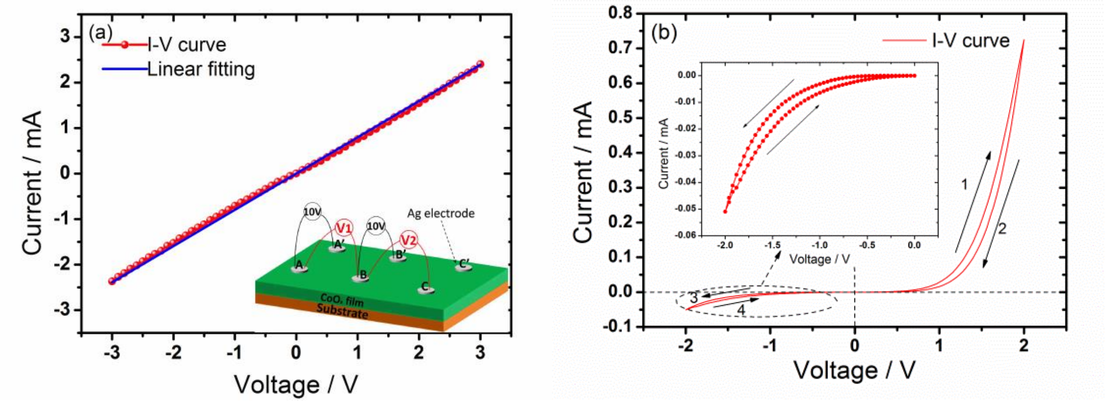

Figure S3│ (a) The linear *I-V* curve measured between electrodes A and B after breaking the interface barriers by applying a voltage of 10V on AA' and BB' electrode pairs. The inset is a schematic of asymmetric interface experiment. (b) The rectifying *I-V* curve measured between electrodes *B* and *C*. The inset is a zoom in image of negative branch of the *I-V* curve.

In order to create asymmetric electrode interfaces, we broke one of the interface barriers by applying a high voltage. As illustrated in the inset of Fig. S3(a), a 10 V voltage was applied between electrode pairs AA' and BB' to break the barriers at the electrode interfaces. Then we measured the *I-V* curve between *A* and *B*. The nearly linear *I-V* curve is shown in Fig. S3(a), which indicates that the interface barriers have been destroyed. This result also confirmed that the hysteresis loop is originated from the interface barriers instead of CoO_x itself. After this, we measured the *I-V* curve between the electrodes *B* and *C*, which has a single Schottky barrier in the circuit. The result is shown in Fig. S3(b). It is a typical rectifying *I-V* curve with a little hysteresis loop. This asymmetric *I-V* curve is corresponding to the asymmetric barrier of electrodes and the resistance of reverse biased barrier is more than one order larger than that of the forward biased one. It supports the assumption that the voltage drops mainly on the reverse biased one when there are both reverse and forward biased barriers in the circuit. So it is easy to understand that the *I-V* curve will be dominated by the property of reverse biased barrier in low voltage range.

From the inset of Fig. S3(b), it can be seen that the negative branch of *I-V* curve is also nonlinear and hysteresis. We analyze the branch 3 of *I-V* curve and find that $\ln(|I|)$ has a linear relationship with $|V|^{1/4}$ and $|V|^{1/2}$ in the voltage ranges of 0~0.8V and 0.8~2V, respectively. This is in agreement with the analysis in the article.

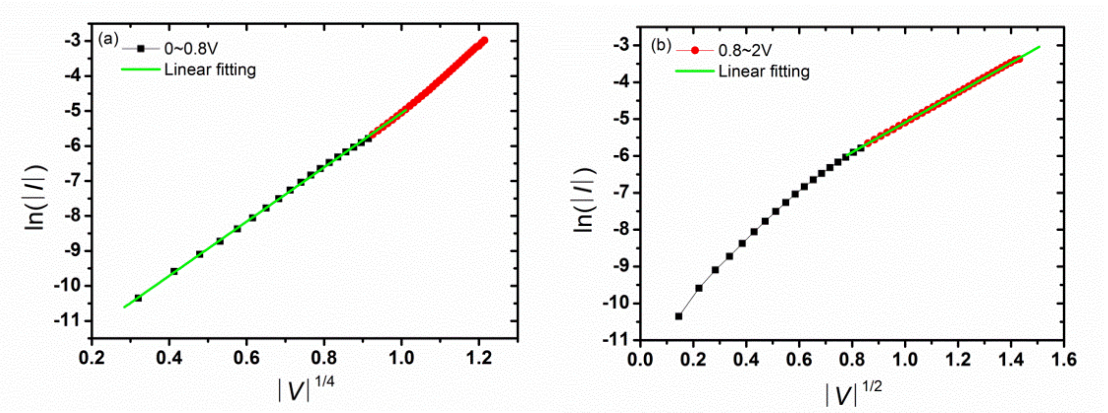

Figure S4│ The linear fittings of $\ln(|I|)$ versus $|V|^{1/4}$ and $|V|^{1/2}$ of the branch 3 of asymmetric *I-V* curve in the voltage ranges of (a) 0~0.8V and (b) 0.8~2V.

IV. Temperature dependent and various oxygen atmosphere behaviors

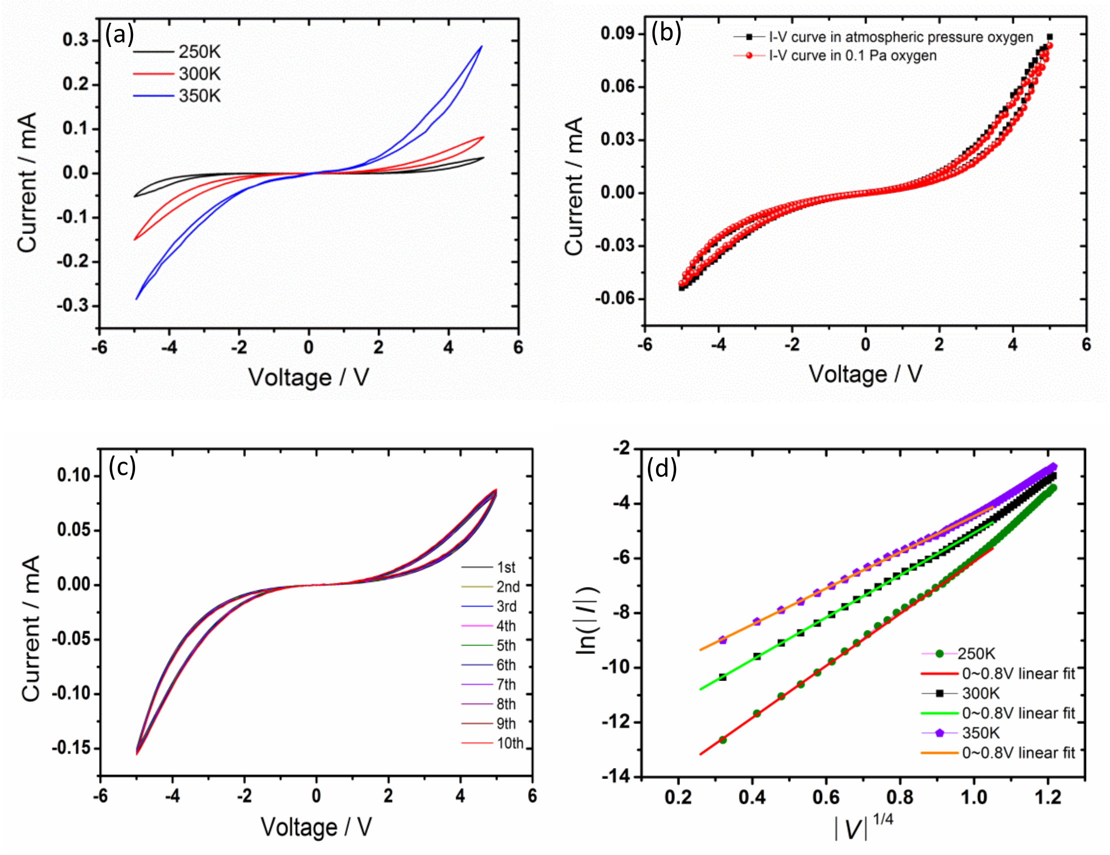

Figure S5│ (a) *I-V* curves under various temperatures. (b) *I-V* curves under various oxygen pressures. (c) Ten times repeated measurement of *I-V* curves under 300K. (d) Partial linear fittings of $\ln(|I|)$ versus $|V|^{1/4}$ of *I-V* curves under different

temperatures.

To explore the stability under different ambient conditions, we measured the electrical properties under various temperatures of 250K, 300K and 350K and the *I-V* curves have been shown in Fig. S5(a). We can see that the currents are intensely increasing with temperature.

Moreover, the *I-V* curves in atmospheric pressure and 0.1 Pa of oxygen in Fig. S5(b) show that there are no obvious differences under various oxygen pressures. This result rules out the possibility of extensive redox.

Also, we repeated the measurement of *I-V* curves at 300K to verify the stability (Fig. S5(c)). It can be seen that ten *I-V* curves overlap together, which shows a highly repeatability under the same condition.

To extend this model to other temperatures, we partially fitted the relationship of $\ln(|I|)$ versus $|V|^{1/4}$ for the *I-V* curves under 250K, 300K and 350K. As shown in Fig. S5(d), the slopes of the fitting curves decrease with the increasing of temperature, which is in agreement with that the slope is proportional to $\frac{1}{T}$ described in Equation (8) in the article.

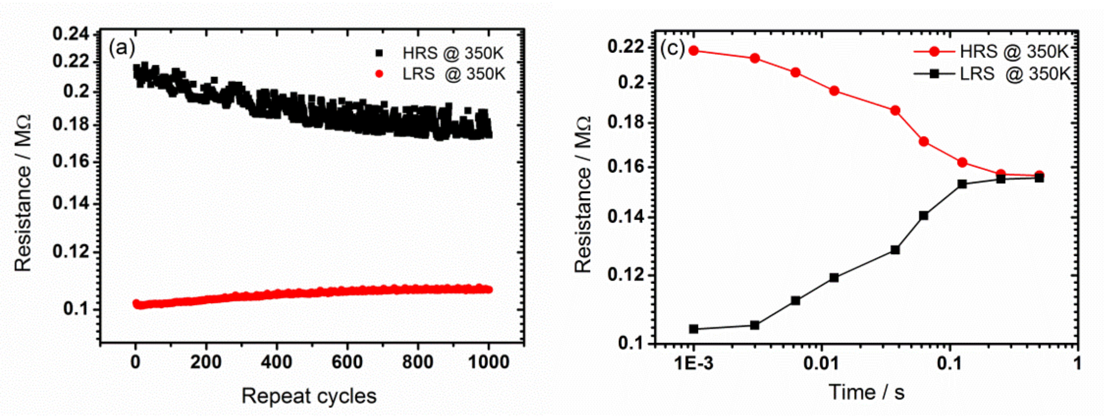

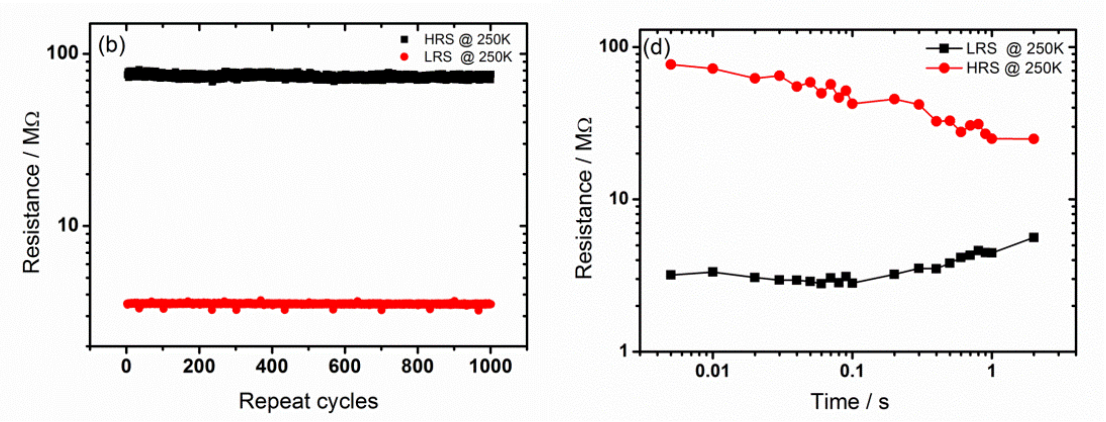

Figure S6│ The results of endurance test at the temperature of (a) 350K and (b) 250K. The first read out resistances of HRS and LRS versus the delay time between the switching voltage and the first reading voltage at the temperature of (c) 350K and (d) 250K. The switching voltages are ±5 V, and the reading voltage is +0.1 V. All y-axis are in logarithmic.

The measurement results of endurance and decay behavior at 250K and 350K are shown in Fig. S6. It can be seen from (a) and (b) that the resistances of HRS and LRS and the switching ratios decrease with the increasing of temperature. From (c) and (d) we can see that the lifetime of HRS and LRS decrease with the increasing of temperature. This supports the view point that the low temperature helps to stabilize the RS while the high temperature aggravates it.

V.Decay process

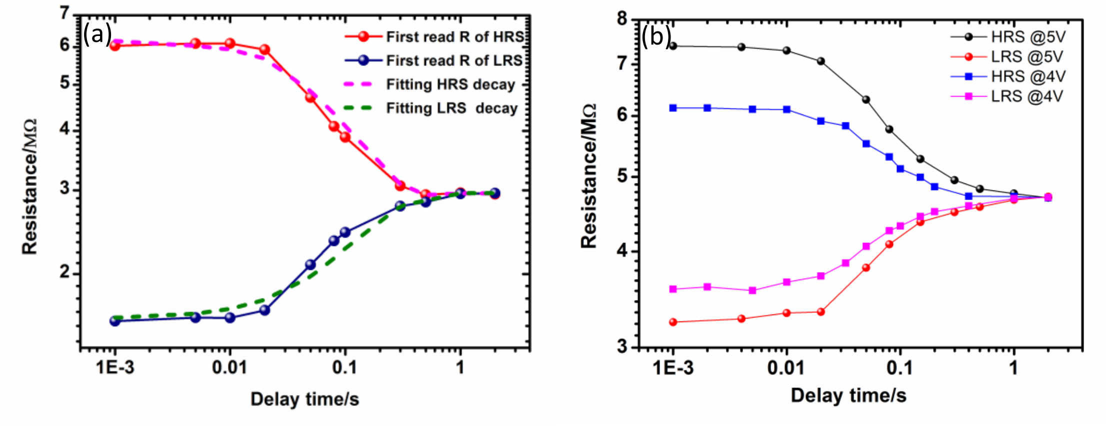

Figure S7│ (a) The magenta and olive dashed line is the fitting curve of HRS and LRS in Fig. 4(a) with Eq. (7) and Eq. (13), respectively. (b) Decay process under the switching voltage of $\pm 5\,\mathrm{V}$ and $\pm 4\,\mathrm{V}$, respectively.

An average lifetime $\tau \approx 0.5$ second and $\beta \approx 0.017$ were obtained from the fitting results in Fig. S7(a). On the other hand, we can roughly verify the fitting parameter τ by reading out the lifetime from the Fig. 4(a).

To explore the effect of different switching voltages on the decay process, we measured the decay processes with the switching voltages of $\pm 5\,\mathrm{V}$ and $\pm 4\,\mathrm{V}$, respectively. Results in Fig. S7(b) indicate that the values of HRS and LRS are dependent on switching voltage, while the decay constant is independent of it. This property allows multi-bit storage in a single memory unit.

Reference

1. Ba, R.B. et al. Synthesis of Co-based bimetallic nanocrystals with one-dimensional structure for selective control on syngas conversion, *Nanoscale*, 7, 12365 (2015).
2. Waser, R., Dittmann, R., Staikov,G. and Szot, K. Redox-Based Resistive Switching Memories – Nanoionic Mechanisms, Prospects, and Challenges. *Adv. Mater.* 21, 2632–2663 (2009).
